# The pattern of romantic and sexual related experiences among Chinese young adolescents: an exploration with multi-group latent class analysis

**DOI:** 10.1186/s12978-021-01235-3

**Published:** 2021-09-20

**Authors:** Chunyan Yu, Chaohua Lou, Qiguo Lian, Xiaowen Tu, Jiashuai Zhang, Xiayun Zuo

**Affiliations:** 1 NHC Key Lab. of Reproductive Regulation (Shanghai Institute for Biomedical and Pharmaceutical Technologies), Fudan University, 779 Old Hu Min Road, 200237 Shanghai, People’s Republic of China; 2 School of Public Health, Fudan University, Shanghai, China

**Keywords:** Romantic and sexual related behaviors, Multi-group latent class analysis, Ecological factors, China

## Abstract

**Background:**

Studies on very young adolescents’ romantic and sexual experiences would help inform the context in which early sex arises. However, such studies are scant in China due to sparse data and cultural issues.

**Method:**

This study used the GEAS baseline data conducted among1776 adolescents in Shanghai. Multi-group latent class analysis was used to explore adolescents’ romantic and sexual experiences patterns and subgroups. Multi-nominal logistic regression was performed to identify the factors distinguishing different subgroups subsequently.

**Results:**

There were gender differences in the lifetime prevalence for very young adolescents’ romantic and sexual-related behaviors. The Multi-group latent class analysis indicated that the participants could be classified into three classes: *general group*, *early romance group*, and sex *exploratory group*. Multi-nominal logistic regression showed youth in the *early romance group* were more likely to had friends of both gender, ever had a romantic relationship, and had more autonomy in deciding where to go than th*e general group*; while male respondents in the *sex exploratory group* were older, ever had a romantic relationship, believed that boys should be more sexually active and more proactive than girls, had more autonomy on deciding where to go, and perceived less school connection and neighborhood cohesion. Female respondents in the *sex exploratory group* were older and less empowered in decision-making than the *general group*.

**Conclusions:**

The result provides a picture of romantic and sexual behavior patterns among both gender of very young adolescents in China. Current sex education needs not only to be culturally appropriate but also to address the harm of gender inequality and stereotypes, as well as to provide accessible and supportive services to help young adolescents personalize their received information and strengthen their skills in communication, decision making, and critical thinking.

## Plain language summary

Studies on teenagers' romantic and sexual experiences would help to prevent early sex arises before it really takes place. However, such studies are limited, especially in China. The traditional culture thought that youth should not start romance before college. This study used the Global Early Adolescent Study Shanghai site baseline data collected among1776 teenagers to explore their romantic and sexual experiences patterns and identify the influencing factors. There were significant gender differences in teenagers’ romantic and sexual-related behaviors. According to the multi-group latent class analysis, we classified the teenagers into three classes: the* general group*, the *early romance group*, and the *sex exploratory group*. The multi-nominal logistic regression showed youth in the *early romance group* were more likely to had friends of both boys and girls, ever had boyfriend/girlfriend and were freer to go out than the *general grou*p; while boys in the *sex exploratory* *group* were older, ever had a boyfriend/girlfriend, believed it was normal for boys to be sexually active than girls, had more freedom to go out, and had less perception of care from school adults and neighborhood. Girls in the *sex exploratory group* were older and less likely to make decisions on their own. Given the result we found, we call for health researchers and educators to conduct sex education programs promoting more equal gender norms and provide services to help young adolescents get right information and strengthen their skills in communication, decision making and critical thinking.

## Background

Sex and reproductive health (SRH) issues affecting adolescents in developing countries have received more and more attention in the recent 30 years since 1994 [1]. There are increases in policies and programs to improve their situation, but most focus on older adolescents. Early adolescence (the years between 10 and 14) is generally considered one of the healthiest life stages [2]. However, their status –dealing with the emerging romantic interest and sex desire (not necessarily being involved in penetrative sex behaviors) in the context of rapid puberty changes – has been overlooked [3]. SRH contributes significantly to the health status of young adolescents even while the majority has not engaged in sexual intercourse. Unsafe sexual behaviors are major contributors to disability-adjusted life years (DALYs) lost for very young adolescents in nearly every region of the world [4]. As young people transition from the early to late adolescent years, sexual and reproductive behaviors contribute to diverging mortality and morbidity patterns by gender, with young girls facing an increased risk of experiencing sexually coercive interactions, contracting STIs (including HIV), as well as suffering the gender-specific consequences of unintended pregnancies and psychological trauma [4].

Studying very young adolescents’ romantic and intimate sexual practices could help inform the context in which most sexual behaviors occur [5, 6]. Researches on adolescents’ sexual trajectories from western and African countries indicated that normative heterosexual behavior among older adolescents develops in an orderly, progressive sequence: from noncoital sexual interactions, such as kissing, hugging, fondling or petting, generally precedes engagement in the next [7–9]. Recently, there is a study in Kenya explored young adolescents’ intimate sexual practices before sexual intercourse through latent class analysis [10]. It identified three subgroups of youth characterized by involving, observing or being naïve to romance, suggesting merely looking at the prevalence of sexual intercourse would miss the point for early prevention. However, most of the above studies were in the US, Europe, or Sub-Saharan Africa. The information on the normative romantic and sexual-related experiences from very young Chinese adolescents is scant.

China is quite different from Western and African countries both in sex behavioral patterns and sex-related norms [7, 11, 12]. Some researchers argue that Chinese populations are strongly influenced by Confucianism and believing that women should stay virgins until they get married, and would rarely think of having sexual intercourse before age 15 [13, 14]. However, significant and rapid social changes have taken place in China’s coastal regions, majorly because of the urbanization and westernization during the past four decades [7, 15]. Attitudes towards romance and sex also changed dramatically [16]. The sexual interest among middle school students is considered normative now [17]. Some middle school adolescents have begun to explore intimate relationships.

The present study is guided by Blum’s conceptual framework regarding early adolescence research based on the ecological model [18]. Young adolescents’ behaviors could be influenced by both the characteristics of individuals (Micro level influences), and the nature of the contexts - such as families, peer groups, schools, and neighborhoods’ environments - within which they socialize, study, and reside (Meso level influences), as well as the policies and norms they were exposed to(Macro level influences) [3, 18]. For instance, a review indicates that older age, early onset of puberty, being male, and using alcohol were some of the key risky individual factors for ever had sex among youth in developing countries [19]. No parental care and less family stability are considered risky for early sex [19]. Related research points out that befriended adolescents are likely to be similar to each other in behaviors and characteristics [20]. Care from school adults is a protective factor for youth in developing health risky antisocial behaviors [21]. Neighborhood disadvantages are correlated with risky adolescent sexual behaviors such as early first intercourse and abortion [21, 22]. Macroly, Interactive media use has become ubiquitous among adolescents [23]. Given the everyday use of smartphones and related technology in adolescents’ lives, it is also essential to include sexually related media use and electronic communication within the macro-context of romantic and sexual development (e.g. watching porn, flirting through social media) [24]. Harmful gender norms, such as sexual double standards (beliefs that grant more sexual freedom to men than to women), are reported to be correlated with more permission for casual sex among adolescent boys in both white and Asian Americans [25]. However, such correlations tend to work through adolescent peer acceptance in different gender [26].

As cultural differences and contextual changes exist in the developmental stage of sexuality [7, 27], it is valuable to explore very young adolescents’ romantic and sexual behavioral patterns in the present context of China. This paper aims to add our knowledge mentioned above using the GEAS baseline cross-sectional data collected among young adolescents ages 10–14 years in a less developed community of Shanghai. We hypothesize that the proportion of romantic and experiences among young adolescents varied among boys and girls while the patterns would be identical at some point but would also be different due to the micro-, meso-, and macro- factors [18, 28].

## Methods

### Study design, participants, and procedure

Data were extracted from the baseline study of the Global Early Adolescent Study(GEAS). GEAS is a 5-year longitudinal study implemented through a collaboration of university and research institutions globally, focusing on gender norms in early adolescents(aged 10–14) and their relation to adolescent health, especially sexual and reproductive health, in disadvantaged urban environments.

The GEAS seeks to estimate the frequency of different events (Being in a romantic relationship, engaging in holding hands, flirting, etc.) among boys and girls, respectively, with sufficient precision. Given the lack of information on romance and related behaviors among youth aged 10–14, we assume a frequency of 50 % and calculated that inclusion of 450 boys and 450 girls would allow a precision of ±4.6 %. Considering the annual attrition rate of 10 % requires the initially enrolled sample of our site to be approximately 1450 adolescents aged 10–14.

A stratified cluster sampling procedure was adopted for the selection of participants in the Shanghai site. Three primary public middle schools in two less-developed sub-districts of the Jing’an district were selected. The STROBE cross-sectional reporting guidelines were used as a checklist to draft the manuscript [29].

All study procedures in Shanghai were approved and monitored for ethics by the Medical Ethical Committee of the Shanghai Institute of Planned Parenthood Research (No. PJ2017-27). Parental consent forms of the study were sent to their parents and then collected through class headteachers. Youth assent was collected before the data collection.

Data were collected through tablets using the Computer-Assisted Self-Interview (CASI) method during November and December in 2017. The fieldwork was implemented with the coordination of key informants from the local teachers’ organization. The eligible students in grades 6th to 8th were organized by their teachers in the class units to independently fill in the electronic questionnaire. In each class, 1–2 trained investigators were present in case the participants need assistance with the tablet using or clarification of the unclear question they encountered. The survey took approximately 40 to 60 min to finish. The tablets were returned after the process and checked by the investigators to ensure that all necessary questions were answered before submission. Each student was compensated for their participation with a small gift valued at 20–30 CNY after the process.

Ultimately 1776 adolescents completed the baseline survey using tablets. We excluded 16 participants who were over 14 years old and 47 respondents who had a high share of missing data. The final analytic sample was 1714 (96.5 % of the selected sample).

### Measurements

#### Romantic and sexual related experiences

We asked “Have you watched pornography before?“ by adding an explanation of “movies or videos that show people’s private parts (genitals) during sexual scenes” to help young adolescents distinguishing them from other romantic scenes like kissing or hugging. Options “rarely”, “sometimes”, and “often” were combined and coded as ever, while “don’t know” and “refuse to answer” were coded as missing. We included ever flirted using social media as another indicator reflecting the romantic or sexual related experiences via media, and ever held hands, ever hugged/cuddled, ever kissed, ever touched/fondled private parts, ever had sexual intercourse/oral sex/anal sex as indicators reflecting the romantic and sexual related experiences of direct contact. Options with either “yes, with a boy”,“ yes, with a girl” or “yes, with both” were coded as ever.

#### Covariates

We included individual factors such as age, gender, personal autonomy on freedom of movement(how often they are allowed to do following things without an adult present: Go to after-school activities; go to a party with boys and girls; meet with friends after school; go to community center/movies/youth center; go to church/mosque/temple or religious center; visit a friend of the opposite gender) and decision-making(be able to make a decision on one’s own regarding following things: what cloth to wear when not in school; what to do during free time; what to eat when not at home; how much education to complete; who can have as friends; decide when and who to marry in the future) [30]. For the above two scales, the mean score of each scale was calculated, with a range from 1 to 4. We then included meso factors like parental monitoring( whether they know my friends’ name, know my performance in school, and know where I am; those who checked yes for all the three items were classified in the higher group, whereas the left was classified into the lower group), peer structure(having friends of both genders or having friends of the same gender), peer drinking behaviors(whether had close friends drink), romantic engagement(ever/never), as well as school connectedness(whether there are adults in schools who care about me) and neighborhood cohesion (people in my neighborhood look out for and help each other, they can be trusted, they know who I am, they care about me; those who checked very true or somewhat true for all the four items were classified in the higher group, where as the left were classified into lower group). For the macro-level factor, we included one aspect of the gender norms that could influence adolescents’ behavior selection: the scale of sexual double standard (SDS) developed by the GEAS research group [31]. A mean score and a median score across items were calculated; participants whose mean score fell below the median were classified in the lower group, whereas those who scored equal to or above the median were organized in the higher group.

### Analysis strategy

We used a person-oriented approach, the exploratory latent class analysis(LCA), as it is well-suited for studying romantic and sexual-related behaviors because of their complex, multidimensional nature [32]. Besides, analyzing the romantic and sexual-related behavior patterns, rather than individual behaviors, can bring additional insight into consideration and find the difference with respect to the influencing factors among each pattern of the behaviors [33]. Multi-group Latent Class Analysis(MLCA) is used because the probability of being in a given class could vary by the observed variable group (in our study, the grouping variable is the gender). In terms of LCA, a solution of classes is optimal when classes are as homogeneous as possible while differences between classes are as large as possible. Classes are added until the model fits the data well. Fifty random sets of starting values were used. Models ranging from 2 to 5 classes were evaluated. Model fit was evaluated with the following commonly-used statistics: Akaike information criterion(AIC), the Bayesian Information Criterion(BIC), sample size adjusted BIC (aBIC), and Entropy values. Small values of AIC, BIC and aBIC, and larger values of Entropy, indicate better model fit. LMR and BLRT indicate if this model is better than the model fitted using the previous one.

This paper’s primary purpose is to use LCA and MLCA through Mplus 7.5 to examine the heterogeneity, or variation, in romantic and sexual related behaviors among the sample and identify different types of subgroups of youths across gender assuming heterogeneity across gender exists. The secondary objective was to examine how the identified subgroups are differentially related to the demographic, environmental, and contextual covariates. Thus, following the LCA, an additional multi-nominal logistic regression was performed among each gender group and the total sample, respectively. The latent categorical variables were regressed on the covariates using Stata SE 15.0.

## Results

### Sample description

A total of 1714 respondents were included in the analysis. Male and female youth were distributed equally(50.8 % vs. 49.2 %). Most adolescents have experienced puberty onset, with females showed a higher percentage than males (93.6 % vs. 87.7 %, *P* < 0.001). Over 80 % of the adolescents were living with both parents. Though not so many, about 17.2 % of male respondents and 10.1 % of female respondents reported ever had engaged in a romantic relationship, with significant gender differences (*P* < 0.001). Table 1 listed the details of the sociodemographic characteristics of the analytic sample.

**Table 1:** Socio-demographics of study population, stratified by gender

		Males (N=871, 50.8%)		Females (N=843, 49.2%)		Total (N=1714)	
		$N/{\bar {X}}$	%/SD	$N/{\bar {X}}$	%/SD	$N/{\bar {X}}$	%/ SD
Micro-level							
Age*	≤12	412	47.3%	442	52.4%	854	49.8%
	13–14	459	52.7%	401	47.6%	860	50.2%
Freedom of move**($\overline{\mathrm{X} }$±SD)	Cont.	2.6	0.8	2.5	0.7	2.5	0.7
Decision-making**($\overline{\mathrm{X} }$±SD)	Cont.	3.3	0.7	3.4	0.7	3.4	0.7
Meso-level							
Parent monitoring***	High	682	78.6%	739	87.9%	1421	83.2%
	Low	186	21.4%	102	12.1%	288	16.8%
Friends of both gender***	Yes	476	59.1%	399	49.9%	875	54.5%
	No	329	40.2%	401	50.1%	730	45.5%
Have friends drink	Yes	23	3.0%	15	2.0%	38	2.5%
	No						
Romantic engagement***	Ever	129	17.2%	77	10.0%	206	13.6%
	Never	621	82.8%	692	90.0%	1313	86.4%
School connectedness	Yes	748	92.4%	711	91.2%	1459	91.7%
	No	62	7.6%	69	8.8%	131	8.3%
Neighborhood cohesion	High	515	62.1%	478	59.4%	993	60.7%
	Low	315	37.9%	327	40.6%	642	39.3%
Macro-level							
Sexual double standard	High.	367	44.8%	521	64.6%	888	54.6%
	Low	452	55.2%	285	35.4%	737	45.4%

Total number of each column within the variable may not sum up to the sample/sub-sample size due to missing or/and refusing to answer. Gender differences are compared, ***P<0.001; **P<0.01; *P<0.05

Table 2 shows the percentages of male and female adolescents utilizing each romantic and sexual related behavior and the comparisons between gender using the chi-square test. The prevalence was relatively low, and gender differences could be observed in the romantic and sexual related behaviors, including watching porn, holding hands with the one you loved, kissing, having the sexual touch, and penetrative sexual behaviors. Because the response rate of ever sexting and ever had penetrative sexual behaviors (sexual intercourse, oral sex and anal sex) were too low, we decided to exclude these items in the latent class analysis.

**Table 2:** Prevalence of each romantic and sexual related behavior

		Males (N=871, 50.8%)		Females (N=843, 49.2%)		Total (N=1714)	
		N	%	N	%	N	%
Ever flirted using social media	Yes	101	13.1	87	11.2%	188	12.2
	No	670	86.9	688	88.8%	1358	87.8
Ever sexting	Yes	8	3.6	5	2.7%	13	3.2
	No	212	96.4	181	97.3%	393	96.8
Ever held hands**	Yes	195	25.8	141	18.7%	336	22.3
	No	561	74.2	612	81.3%	1173	77.7
Ever hugged/cuddled	Yes	90	11.8	71	9.3%	161	10.6
	No	671	88.2	692	90.7%	1363	89.4
Ever kissed*	Yes	33	4.3	18	2.3%	51	3.3
	No	730	95.7	753	97.7%	1483	96.7
Ever had sexual touch/fondled*	Yes	38	6.0	19	3.0%	57	4.5
	No	597	94.0	617	97.0%	1214	95.5
Ever watched porn*	Yes	213	25.6	167	20.3%	380	23.0
	No	618	74.4	654	79.7%	1272	77.0
Ever had sexual intercourse**	Yes	9	1.4	0	0.00%	9	0.9
	No	621	98.6	620	100%	1241	99.3
Ever had oral sex*	Yes	12	5.4	3	1.5%	15	3.5
	No	209	94.6	204	98.5%	413	96.5
Ever had anal sex**	Yes	9	3.3	0	0.00%	9	1.8
	No	263	96.7%	234	100%	497	98.2

Total number of each behavior may not sum up to the sample/sub-sample size due to missing or/and refusing to answer

Gender diffences are compared, ***P<0.001; **P<0.01; *P<0.05

### Identifying the latent class of romantic and sexual related experiences

**Fig. 1:**
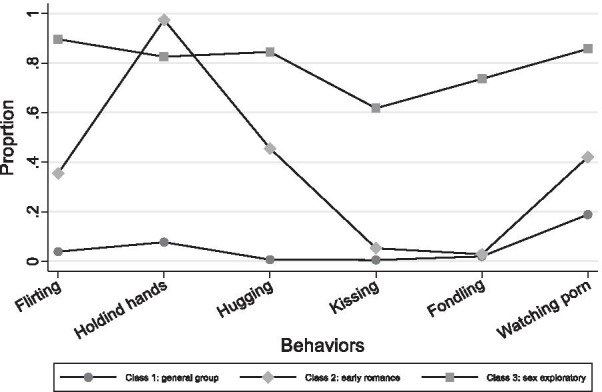
Male group: behavioral patterns based on 3-class multi-group LCA model

**Fig. 2:**
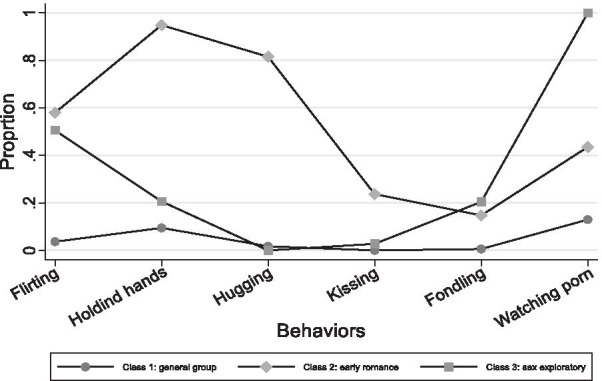
Female group: behavioral patterns based on 3-class multi-group LCA model

We compared models with 2 through 5 latent classes in both LCA models and MLCA models to determine the optimal latent class solution. Table 3 shows the result of the latent class analysis. The lowest AIC and aBIC was research in the 4-class LCA model, while the lowest BIC was reached in the 3-class LCA model. Latent class analysis with the grouping variable was used to examine the invariance in the latent class structured by gender. Similarly, the lowest AIC and aBIC was research in the 4-class MLCA model, while the lowest BIC was reached in the 2-class MLCA model. Taking the Entropy into consideration, the highest Entropy is in the 2-class multi-group LCA model (0.93), while the Entropy for 3-class and 4-class MLCA model is 0.91 and 0.88, respectively. Thus, we decided to select the 3-class MLCA model as the final best-fitting model. Figures 1 and 2 showed the behavioral patterns among male and female groups based on the 3-class multi-group LCA model, respectively.

**Table 3:** Model fit criteria of LCA with an increasing number of classes

N	k	G^2^/LL	Df	Chi2	AIC	BIC	aBIC	Entropy	LMR	BLRT
2	13	163.4	50	223.6	5577.0	5647.8	5606.5	0.86	<0.001	<0.001
3	20	90.4	43	90.7	5518.1	5526.9	5563.4	0.73	<0.001	<0.001
4	27	37.7	36	29.8	5471.5	5618.5	5532.7	0.82	<0.001	<0.001
5	42	22.7	29	36.3	5478.4	5663.5	5555.4	0.90	0.05	0.33

Multi-group LCA										
2	26	209.6	101	311.1	7951.0	8092.6	8005.9	0.93	–	–
3	39	120.7	88	161.1	7888.1	8100.4	7976.5	0.91	–	–
4	52	55.8	75	112.6	7849.2	8132.2	7967.0	0.88	–	–
5	65	36.4	62	37.7	7857.1	8210.9	8004.4	0.91	–	–

Table 4 depicted the observed class memberships and the romantic and sexual related experiences’ endorsement frequencies in the best fitting LCA solution. Table 5 listed the item-response probabilities indicating the likelihood of individuals in each latent class engaging in a particular behavior; the probabilities help interpret the classes produced by multi-group LCA. For example, Class 1 of both male and female were characterized by very low endorsement rates for most of the 6 sex and romance-related behaviors, thus the group of both boys and girls was termed as *t*he *general group*. Class 2 of both male and female were characterized by endorsing romance-related behaviors such as flirting, holding hands, and cuddling. Thus the group of both boys and girls was termed as the *early romance group*. Class 3 of male and female showed a different behavioral pattern: the male group was characterized by a very high endorsement of all the romantic and sexual related behaviors while a higher endorsement of pornography-watch characterized the female group. This group of both boys and girls was termed as the *sex exploratory group*.

**Table 4:** Latent class membership by gender

	Class 1			Class 2			Class 3		
	Male (n=746)	Female (n=734)	Total (n=1480)	Male (n=82)	Female (n=85)	Total (n=167)	Male (n=39)	Female (n=22)	Total (n=61)
Ever flirted using social media	23 (3%)	18 (2%)	41 (3%)	43 (52%)	51 (60%)	94 (56%)	35 (90%)	18 (82%)	53 (87%)
Ever held hands	85 (11%)	61 (8%)	146 (10%)	79 (96%)	80 (94%)	159 (95%)	31 (79%)	0 (0)	31 (51%)
Ever hugged/cuddled	4 (1%)	10 (1%)	14 (1%)	53 (65%)	61 (72%)	114 (68%)	33 (85%)	0 (0)	33 (54%)
Ever kissed	3 (0)	0 (0)	3 (0)	6 (7%)	17 (20%)	23 (14%)	24 (62%)	1 (0)	25 (41%)
Ever had sexual touch/fondled	9 (1%)	3 (0)	12 (1%)	2 (2%)	11 (13%)	13 (8%)	27 (69%)	5 (23%)	32 (52%)
Ever watched porn	148 (20%)	106 (14%)	254 (17%)	31 (38%)	39 (46%)	70 (42%)	34 (87%)	22 (100%)	56 (92%)

N(%) of participants with specific behavior within the class were shown in the table.

**Table 5:** Item-Response Probabilities (Standard Errors) for 3-class MLCA model of romantic and sexual behaviors

			Latent class			
	Class 1		Class 2		Class 3	
	Male (n=746)	Female (n=734)	Male (n=82)	Female (n=85)	Male (n=39)	Female (n=22)
Class Membership Probabilities	0.44	0.43	0.05	0.05	0.02	0.01
Behavioral Indicators						
Ever flirted using social media	0.04 (0.01)	0.03 (0.01)	0.40 (0.09)	**0.58 (0.07)**	**0.88 (0.08)**	0.42 (0.30)
Ever held hands	0.10 (0.02)	0.09 (0.01)	**1 (<0.01)**	**0.95 (0.04)**	**0.82 (0.09)**	0.11 (0.30)
Ever hugged/cuddled	0.01 (<0.01)	0.02 (0.01)	**0.54 (0.11)**	**0.75 (0.14)**	**0.83 (0.11)**	0 (<0.01)
Ever kissed	0.01 (<0.01)	<0.01 (<0.01)	0.06 (0.04)	0.22 (0.06)	**0.59 (0.13)**	0.02 (0.02)
Ever had sexual touch/fondled	0.02 (0.01)	0.01 (<0.01)	0.03 (0.04)	0.15 (0.06)	**0.71 (0.12)**	0.16 (0.16)
Ever watched porn	0.19 (0.02)	0.12 (0.03)	0.42 (0.06)	0.45 (0.09)	**0.85 (0.09)**	**0.99 (<0.01)**

Item-response probabilities greater than 0.5 are bolded to facilitate interpretation

### Latent class membership comparison on covariates

A multi-nominal logistic regression was performed to assess covariates’ influence in the predicted membership compared to the *general group* in the gender-specific sample and the total sample. As shown in Table 6, a number of significant effects were found in the covariates. Among males, youth in the *early romance group* were significantly more likely to have friends of both gender, had friends who drink, ever had a romantic relationship, and had more autonomy in deciding where to go, than the *general group*. In contrast, male youth in the *sex exploratory group* were older, ever had a romantic relationship, perceived more sexual double standards and had more autonomy in deciding where to go, while perceived less school connection and neighborhood cohesion. Among females, youth in the *early romance group* were significantly more likely to report having friends of both gender, ever being in a romantic relationship, and having more autonomy in deciding where to go, similar to that group of boys. Female youth in the *sex exploratory group* were of a different story: they were older and less empowered in decision-making.

**Table 6:** Covariate predictors of latent class membership across gender (comparing with class 1-general group)

		Males		Females		Total	
RRR(95%CI)		2 vs 1	3vs1	2vs1	3vs1	2vs1	3vs1
Age	Cont.	1.4 (0.9–2.1)	1.8 (1.0–3.0)	0.9 (0.6–1.3)	3.0 (1.3–7.0)	1.1 (0.9–1.4)	1.7 (1.1–2.6)
Parent monitoring	High vs. low	1.0 (0.4–2.7)	2.2 (0.5–8.8)	0.7 (0.3–1.9)	0.5 (0.1–2.5)	0.9 (0.5–1.7)	1.2 (0.5–3.1)
Friends of both gender	Yes vs. no	4.1 (1.3–13.2)	3.2 (0.6–15.9)	3.6 (1.5–8.7)	0.6 (0.1–2.2)	3.5 (1.8–7.0)	1.2 (0.5–2.9)
Peer Drink	Yes vs. no	3.02 (1.4–6.6)	2.7 (0.8–8.3)	1.7 (0.8–3.7)	1.4 (0.3–6.1)	2.2 (1.3–3.7)	1.9 (0.8–4.2)
Romantic engagement	Ever vs. never	14.3 (6.5–31.7)	128.2 (28.4–578.2)	6.0 (2.8–12.8)	2.7 (0.5–13.6)	8.3 (4.9–13.9)	21.2 (9.3–48.2)
Scores of SDS	High vs. low	0.7 (0.3–1.6)	4.4 (1.4–13.6)	1.2 (0.6–2.4)	0.5 (0.1–1.6)	1.0 (0.6–1.6)	1.7 (0.8–3.6)
School connectedness	Yes vs. no	0.8 (0.2–2.8)	0.2 (0.04–0.6)	0.7 (0.2–2.1)	0.4 (0.1–1.6)	0.7 (0.3–1.6)	0.28 (0.1–0.7)
Neighborhood control	High vs. low	0.8 (0.3–1.8)	0.2 (0.1–0.7)	0.5 (0.3–1.1)	0.6 (0.2–2.6)	0.6 (0.4–1.1)	0.4 (0.2–0.9)
Freedom of move	Cont.	3.0 (1.7–5.5)	3.5 (1.6–7.8)	1.9 (1.1–3.3)	1.8 (0.7–4.7)	2.1 (1.5–3.1)	2.3 (1.4–4.0)
Decision-making	Cont.	1.29 (0.7–2.5)	0.9 (0.4–2.1)	0.9 (0.5–1.5)	0.4 (0.2–0.8)	1.0 (0.6–1.5)	0.6 (0.4–1.0)

## Discussion

We used cluster samples of young adolescents aged 10–14 in three middle schools of Shanghai to explore the patterns of romantic and sexual-related behaviors distributed in this age group. Unlike most prior work investigating a single sexual behavior, the present study used a multi-group latent class analysis to examine intricate patterns of the sexual and romantic experience. We also examined the factors correlated with different memberships in the latent class. The findings suggest that though most young adolescents have not started any romantic and sexual experiences yet, it is a natural process that needs more attention.

In the membership classification, we found that compared to the *general group*, individuals in the *early romance group* were less likely to experience kissing and fondling but were more likely to experience holding hands and hugging. In western countries, kissing is a sign of a relationship and serves as relationship maintenance and potential romantic partners’ assessment for further sexual attachment [34]. However, despite widespread acceptance that dating is becoming increasingly popular among adolescents, kissing and fondling are still somewhat taboo in Chinese culture [7]. While there is no clear definition of an appropriate age for individuals to begin a romance, those who showed their sexual interest at early ages will typically have to go against the adults [35]. Not surprisingly, Chinese parents and teachers tend to discourage young adolescents from becoming sexually active. Many adults are opposed to adolescent students being involved in relationships until they enter college. It might explain why we found that perceived no care from school adults was associated with the *sexual exploratory group*, and adolescents being classified in *early romance* and *sexual exploratory group* perceived more freedom of movement (autonomy of deciding where to go).

Another aim of this study was to determine if there was a similar pattern of sex and romantic experiences among very young adolescent boys and girls. The findings suggested gender differences in their romantic and sexual behavior patterns as revealed in adolescents from other Asian countries [36]. While in the sex exploratory stage, males tend to experience all the possible ways to explore their sex interests; Females showed an entirely different tendency. For instance, girls were more reluctant to explore this sex interest through the interaction with the boys; instead, they resort to pornography to satisfy their curiosity about sex. The high rate of pornography use and flirting through social media but the absence of kissing and fondling in the girls’ *sex exploratory* latent class group was noteworthy. Kissing and fondling are always relationally or romanticly meaningful. Therefore, the absence of kissing may connotate a lack of affection or emotional intimacy in this group. It echos the multi-group logistic regression results that the covariate romantic engagement was not significantly associated with this group compared with the *general group*. In the Chinese culture, where the Confucian philosophy is valued, women are expected to maintain the three rules of obedience: (1) obeying their fathers and brothers prior to marriage, (2) obeying their husbands within marriage, and (3) as a widow, obeying their adult sons [12]. This set of beliefs, while seemingly outdated in contemporary society, nonetheless still has its impact.

Indeed, several studies have suggested that even in the face of modernization and Western culture’s influence, traditional gender attitudes may persist. Sexual double standards often refer to notions that people judge sexual behavior differently depending on whether the sexual act is a woman or a man. With traditional sexual double standards, it is more socially acceptable for men to engage in sexually permissive behavior. At the same time, women tend to receive stigma for expressing or pursuing their sexual desires [25]. Past research on female sexual development reveals that sexually active girls always are considered as “bad girl” or “sluts”, while sexually non-active girls are thought as “good girls” or “virgins" [37, 38]. The social changes of China, such as the urbanization and migration, which brought the income growth of citizens and the popularity of consumer culture, create conditions for materializing the female [39]. With the widespread use of mass media platforms (TV, film, internet, etc.) and personal communication devices such as smartphones, the notion of sexual attractiveness and the obedience of females are strengthened by media [11]. Good girls are expected to be not sexual and then deserving of respect. Adolescents are highly vulnerable and susceptible to the information conveyed by the pervasive media. Thus, female adolescents might feel expected to prove or seek attractiveness through online activities or relatively private activities (such as watching pornography) while simultaneously managing the perception of few sexual and romantic behaviors offline. Echoing the influence of stereotypical gender attitudes imposing on girls, we also found a significantly higher sexual double standard score among the boys in the *sex exploratory group*. According to social structural theory, the gendered division of labor and gender difference in power place men in a dominant role and women in a subordinate role [40]. Boys are expected to be more sexually active and show their prowess through more sexual experiences. That might explain why we see the more apparent differences in romantic and sexual experiences between gender.

Sexual Scripting Theory would suggest that people are sexually socialized throughout the script (behaviors, concepts, norms, or situations) they perceived. Previously conducted studies indicated that female adolescents and emerging adults who use pornography tend to have more sex partners, report copying behaviors seen in pornography, and engage in sexual behaviors at a younger age [41]. Our result showed that girls in the *“sex exploratory”* group were more likely to watch pornography. It may signal a critical intervention point for health researchers and educators. In addition, female adolescents may benefit from sex education programming that addressing gender equality and empowerment and promoting critical thinking skills for girls to mitigate downside effects from pornography viewing.

Despite providing insights into the classification of romantic and sexual related behaviors among very young adolescents living in Shanghai’s urban-poor setting, our study also provides some implications for intervention: First is cultural appropriateness, for instance, in the norms of Chinese adolescents, the freedom of movement means the autonomy of deciding where to go literally, which might be quite different of what Western or African adolescents think of as the freedom to have multiple romantic/sexual relationships/partners. Thus we should consider the cultural background when looking at the risk factors. Second, future interventions and sex education should target young adolescents aged 10–14 to mitigate the pervasive influence of stereotyped gender attitudes in sex development. Third, since peers and the media place more and more impact on youth, it is essential to equip them with the comprehensive knowledge and skill on sex and relationship as well as providing necessary caring and services: not only the knowledge about the biological differences of male and female, but also teaching them about how to see themselves and others as equal members in their relationships, the ability to protect their health, and as individuals, be capable of engaging as active participants in society [42]. Besides, it is also essential to acknowledge that it is normative for adolescents to be curious about sex and seek out sexual information or seek interaction online and offline. Therefore, experiencing romance and sexual behaviors should not be pathologized.

This paper is not without limitations. First, we used self-reported data in the analyses, which could be biased as previous studies indicated that boys might overreport their sexual behaviors while girls tend to underreport such behaviors. Second, although we included several indicators shown in the literature to assess the patterns of adolescents’ romantic and sexual-related behaviors, additional components regarding feelings were not included (such as sexual fantasies, positive/negative feelings towards intimacy). Future works could examine how such uncovering factors inform group identification for better-tailored intervention. Third, the study used an urban poor, non-representative sample to analyze, which prevents its generality to a broader range of adolescents in rural areas or country levels. However, it does show some unique characters of Chinese adolescents as the sexual scripts in China are quite different from western or African countries. Finally, as classes revealed gender and other ecological factor differences, researchers should carefully select covariates that were important to their research questions when planning the LCA analyses. There could also be other protective mediating or moderating factors not included. Nonetheless, this study’s classified behavioral patterns helped identify the potential indicators of an increased risk for very young adolescents in China.

## Conclusions

This study used a data-driven approach to reveal the romantic and sexual-related behavior patterns among very young adolescents. Our analysis showed three behavioral patterns using gender as a grouping variable: *general, early romance, and sex exploratory*. More disparities and different influencing factors were found in the *sex exploratory* group between boys and girls. Current sex education needs not only to be culturally appropriate but also to address the harm of gender inequality and stereotypes, as well as to provide accessible and supportive services to help young adolescents personalize their received information and strengthen their skills in communication, decision making and critical thinking.

## Data Availability

The datasets used and analyzed during the current study are available from the corresponding author on reasonable request.

## Abbreviations

GEAS: The Global Early Adolescent Study
SDS: Sexual Double Standard Scale
LCA: Latent class analysis
MLCA: Multi-group latent class analysis
